# The design, implementation, and effectiveness of intervention strategies aimed at improving genetic referral practices: a systematic review of the literature

**DOI:** 10.1038/s41436-021-01272-0

**Published:** 2021-08-24

**Authors:** April Morrow, Priscilla Chan, Katherine M. Tucker, Natalie Taylor

**Affiliations:** 1 The Daffodil Centre, The University of Sydney, a joint venture with Cancer Council NSW, Sydney, NSW Australia; 2 Faculty of Medicine and Health, University of Sydney, Camperdown, NSW Australia; 3 Hereditary Cancer Clinic, Prince of Wales Hospital and Community Health Services, Randwick, NSW Australia; 4 UNSW Prince of Wales Clinical School, Randwick, NSW Australia

## Abstract

**Purpose:**

Despite rapid advancements in genetics and genomics, referral practices remain suboptimal. This systematic review assesses the extent to which approaches from implementation science have been applied to address suboptimal genetic referral practices.

**Methods:**

A search of MEDLINE, EMBASE, and PsycINFO generated 7,794 articles, of which 28 were included. Lay barriers were mapped to the Theoretical Domains Framework (TDF) and interventions mapped to behavior change techniques. Use of implementation and behavior change frameworks was assessed, and the Theory and Techniques Tool used to determine theoretical alignment.

**Results:**

Knowledge was the most frequent retrospectively TDF-coded barrier, followed by environmental context and resources, and skills. Significant referral improvements occurred in 56% of studies. Among these, the most frequent interventions were clinical data review systems, family history collection and referral tools, and embedding genetics staff into nongenetic specialties. Few studies used implementation frameworks or reported implementation outcomes, though some deployed intuitive strategies that aligned with theory.

**Conclusion:**

Genetic referral interventions are rarely informed by implementation and/or behavior change theories, limiting opportunities for learning across contexts. Retrospective coding has provided a suite of theoretically linked strategies, which may be useful for informing future efforts. Incorporating these strategies into clinical guidelines may facilitate operationalization within the system.

## INTRODUCTION

Rapid advances in genetic and genomic research have promised to transform future approaches to disease prevention, detection, and treatment [1]. The development of high-throughput technologies for genetic sequencing and analysis has generated new opportunities for improved diagnosis of genetic disorders, personalized targeted treatments (particularly for cancer patients), prenatal screening and diagnosis, and pharmacogenomics [2]. For this research to be clinically translated, health-care professionals must first be able to identify patients who would most benefit from genetic assessment, then facilitate the necessary referral pathways to ensure equitable access and uptake.

However, a wealth of evidence highlights suboptimal genetic referral rates across a range of clinical settings. In the cancer setting for example, studies have demonstrated referral rates of less than 30% among patients at high risk of Lynch syndrome (e.g [3]). Similar findings are demonstrated in hereditary breast and ovarian cancer, with referral rates among eligible patients of less than 50% (e.g., [4]). Given the well documented health benefits of risk management protocols for those affected by hereditary cancer syndromes, these studies signify missed opportunities for cancer prevention, treatment and early detection. For example, preventable (and often incurable) *BRCA1/2*-related cancers have been reported in families where relatives with previous breast and/or ovarian cancer were never referred for variant testing, despite meeting clinical criteria [5]. Beyond the cancer setting, suboptimal referral practices have also been demonstrated across other genetic specialties (e.g., prenatal risk assessment [6], hereditary cardiac conditions [7]). A recent scoping review identified that up to 58% of nongenetics health-care professionals (across various specialties) had never referred a patient for clinical genetics assessment [8].

A number of studies have explored barriers to genetic referral practices, both at the patient and health-care provider levels [9]. At the patient level, barriers include lack of awareness about personal risk and/or family history, and lack of knowledge of genetic services [10, 11]. At the health-care provider level, barriers include lack of knowledge about genetic conditions and patient risk factors, inadequate family history documentation, lack of awareness of genetic services, inadequate referral coordination, and genetic workforce issues [9, 12, 13]. Developing an in-depth understanding of barriers is necessary for the design of targeted strategies to overcome them. The Theoretical Domains Framework (TDF) [14] synthesizes a range of behavior change theories to facilitate the identification of behavior change determinants (e.g., barriers and/or facilitators). Use of a theoretical framework can facilitate the identification of more complex, individual-level barriers (e.g., emotion, social influences) that are less likely to be articulated through standard (non–theory informed) approaches, thereby enabling more comprehensive barrier assessment [15]. However, the extent to which genetic referral barriers have been assessed or classified using theoretical approaches is unknown.

Implementation interventions involve strategies designed to change behaviors at the organizational, provider, or patient level to enhance the adoption of a given clinical intervention or practice [16]. In the context of genetic referral practices, multiple behaviors are involved in the processes through which health-care professionals identify patients who warrant genetic assessment and/or testing (e.g., eliciting family history, interpreting risk, applying referral guidelines) and initiate the referral (e.g., discussion with patient, identifying the appropriate genetic service, writing the referral letter). In this setting, behavior change interventions, underpinned by existing psychological theories, may be effective in achieving clinical practice change [17]. Behavior change techniques (BCTs) are the “active ingredients” of an intervention with the potential to change behavior, while mechanisms of action refer to the processes through which BCTs produce their effects [18]. The TDF can facilitate the identification of behavior change determinants, which can then inform the design of intervention strategies that employ BCTs with known mechanistic links.

Despite the potential benefits, these frameworks are currently underutilized in health-care improvement efforts [19]. While health-care providers are ideally placed to apply their tacit and contextual knowledge to intuitively develop strategies to address the clinical problem they are attempting to solve (“informal theory”), explicitly stating the underlying theoretical causal assumptions can enhance opportunities for learning, replication and generalizability across other settings. Although the ideal is to use theory prospectively, retrospectively determining the extent to which intuitive strategies are represented by BCTs with established mechanistic links (i.e., theoretical alignment) may help to optimize the design of prospective intervention strategies, while developing an understanding of the processes through which these strategies produce their effects [20]. This also improves standardization and generalizability, enabling adaptation of interventions to other contexts while keeping active ingredients constant.

Even when interventions are carefully designed, success is dependent on how well those interventions are implemented in practice [21]. Process evaluations are exploratory studies that seek to complement outcome evaluations by understanding how an intervention works in practice, and are crucial in distinguishing between interventions that are inherently faulty in concept or design, versus those that are poorly implemented. The UK Medical Research Council (MRC) highlights the need for process evaluations alongside complex interventions to assess implementation outcomes (e.g., the quality and quantity of what is delivered), clarify causal mechanisms, and identify contextual factors—all of which can be associated with variation in clinical and/or service level outcomes [22].

The extent to which these approaches from the field of implementation science have been applied in the genetics setting to address suboptimal referral practices is unknown. To address these gaps, the aims of this review were to:

1. Describe health-care provider interventions aimed at improving genetic referral practices, and assess their impact
2. Retrospectively code barriers and intervention strategies against a theoretical framework of behavior change, and assess evidence of potential mechanistic links
3. Assess the extent to which implementation science theories and frameworks have been applied in the design of interventions
4. Determine the extent to which process evaluation and implementation outcome data has been collected to explain clinical intervention outcomes

## MATERIALS AND METHODS

The protocol for this systematic review was prospectively registered through PROSPERO (CRD42020166632) and findings have been reported in line with Preferred Reporting Items for Systematic reviews and Meta-Analyses (PRISMA) statement [23].

### Search strategy

Using database-specific subject headings, MEDLINE (including Medline Epub Ahead of Print, In-Process & Other Non-Indexed Citations), EMBASE, and PsycINFO were searched. Search terms for genetic counseling and testing were combined with referral terms (see Supplementary File 1). The search was limited to articles published in English after 1 January 2000 (as older studies are unlikely to be relevant to current practice). Monthly auto-alerts were generated and continued until completion of data extraction. Search results were exported into Endnote X9 (Thomas Reuters) for screening against inclusion and exclusion criteria. For included studies, reference lists were screened for additional relevant studies. The final database search was performed on 1 May 2021.

### Inclusion and exclusion criteria

Inclusion and exclusion criteria were developed and refined using the PICOS framework (see Supplementary File 2). Articles were included if they:

1. Described an implementation intervention aimed at improving genetic referral practices by targeting a health professional group or health service/system;
2. Reported clinical outcome data relevant to genetic referral practices (e.g., genetic referral rates and/or surrogate outcomes, such as genetic counseling attendance or genetic testing uptake) with a relevant comparator (e.g., preintervention baseline, standard care, other implementation intervention);
3. Were based in Australia, New Zealand, Europe, Canada, the United States (given similarities in health service settings and genetic testing and referral guidelines); and
4. Were published in English in a peer-reviewed journal between 2000 and 2021.

Articles were excluded if they:

1. Described a technical intervention (e.g., introduction of new clinical processes without any accompanying implementation components) or mainstreaming intervention (e.g., provision of genetic counseling or testing by nongenetics health-care professionals);
2. Described an implementation intervention targeted specifically to nonclinical, sociodemographic subgroups (due to limited generalizability); or
3. Were conference proceedings, protocols, case studies, commentaries, letters, editorials, or scoping/narrative reviews.

### Screening and data extraction

**Fig. 1:**
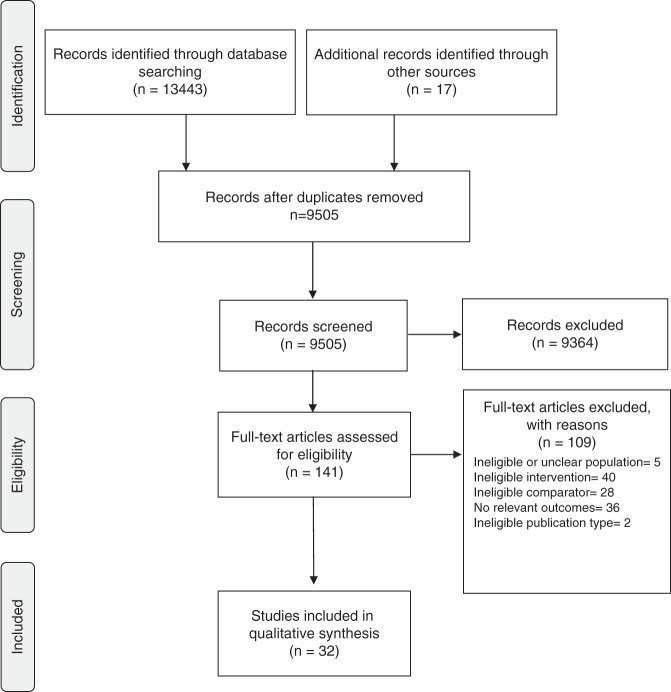
PRISMA flow diagram. Following deduplication, 9505 articles were assessed against eligibility criteria, resulting in 32 articles for inclusion.

Titles and abstracts were screened against inclusion/exclusion criteria by a single reviewer (A.M.—reviewer 1; see Fig. 1.). Articles that were potentially relevant (or of unclear relevance) were selected for full-text review. A prespecified inclusion/exclusion form was independently piloted by reviewer 1 (A.M.) and reviewer 2 (P.C.) using the first ten articles. Following review and refinement of the form, all full-text articles were independently assessed by reviewer 1 and reviewer 2. Discrepancies were resolved via discussion, with adjudication by a third reviewer (N.T.) where consensus could not be reached. For articles with missing or unclear information, authors were contacted for clarification. For all excluded articles, reasons for exclusion were documented.

An initial data extraction form was developed to address the research aims, and to include key items from published intervention reporting guidelines—the Standards for Reporting Implementation Studies (StaRI) statement [24] and Template for Intervention Description and Replication (TIDieR) checklist [25]. These guidelines have been developed to ensure adequate description of implementation studies and intervention strategies to enhance replicability. The extraction form was piloted by two reviewers (A.M. & P.C.), with discussion and refinement among the research team. Reviewer 1 extracted data from all studies selected for inclusion, and reviewer 2 independently extracted data for 20% of randomly selected studies to ensure accuracy.

### Data analysis

Where possible, reported barriers to genetics referral were mapped to the TDF [14]. The TDF is a validated framework that can be used to classify barriers according to theoretically underpinned psychosocial domains of behavior change, and has been extensively used across a range of clinical settings [26]. Implementation strategies were coded to a refined taxonomy of 73 discrete strategies identified through the Expert Recommendations for Implementing Change (ERIC) project in response to the need for a consistent nomenclature for implementation terms and definitions [27].

Intervention content descriptions were further mapped to the BCT Taxonomy version 1 [28], which consolidates a large number of published intervention components designed to alter causal processes influencing behavior (e.g., the “active ingredients” of an intervention). Studies were further categorized according to the extent to which theory and/or implementation frameworks were applied in intervention design, using the theoretical coding scheme developed by Michie and colleagues [29].

Where barriers were described in sufficient detail to allow mapping to TDF domains and BCTs were identified in subsequent intervention components, the Theory and Techniques Tool [30] was used to determine evidence of theoretical alignment (i.e., mechanistic links) between the BCTs used to target TDF-mapped barriers. The Theory and Techniques Tool is an interactive online resource consolidating links between BCTs and their mechanisms of action based on a synthesis of published intervention studies and expert consensus study [30]. Process evaluation and implementation outcomes were coded against the framework by Proctor et al. [21] that consists of eight implementation outcomes (acceptability, adoption, appropriateness, feasibility, fidelity, implementation cost, penetration, and sustainability), which are conceptually distinct from clinical and service system outcomes. All coding was performed by one author (A.M.), with 40% independently coded by a second author (N.T.) to ensure agreement. Coding for all remaining articles was reviewed by N.T. Both A.M. and N.T. have prior experience coding intervention content using these frameworks.

Extracted and coded data were synthesized using a narrative framework (incorporating theoretical perspectives), with findings tabulated, grouped, and structured into key themes by one author (A.M.), with discussion and refinement among the wider team (P.C., N.T., K.M.T.).

### Quality assessment

The quality of all articles were assessed using QualSyst, which accommodates assessment of both qualitative and quantitative study designs simultaneously [31]. The quality appraisal was conducted by a single reviewer (A.M.) and checked by an additional reviewer (P.C.). Studies were not excluded on the basis of quality; however, quality was considered in the interpretation of findings. Missing data were factored into the quality scores. A Grading of Recommendations Assessment, Development and Evaluation (GRADE) assessment was performed to assess quality across studies and certainty of the evidence [32].

## RESULTS

### Study characteristics

Thirty-two studies were included in the review, the characteristics of which are summarized in Supplementary File 3. Most studies took place in the hospital setting (*n* = 26; 81%), and in the context of hereditary cancer (*n* = 29; 91%). Most described cohort studies (*n* = 26; 81%), while the remaining were cluster randomized controlled trials (*n* = 5; 16%) or stepped-wedge trials (*n* = 1; 3%). Over half took place in the United States (*n* = 20; 63%).

### Referral barriers

Twenty-four articles (75%) explicitly stated barriers to genetic referral, which were mapped to eight TDF domains [14]. Knowledge was the most frequently cited referral barrier (*n* = 16 studies; 67%), followed by environmental context and resources (*n* = 10, 42%), skills (*n* = 8, 33%), and memory, attention and decision processes (*n* = 8, 33%). Table 1 provides details of the barriers cited within each domain. In the majority of articles (*n* = 21; 88%) barriers were identified from published literature or anecdotal reports, whereas three studies (13%) conducted formal assessment of local barriers (see “Implementation strategies” below). One study [3] explicitly classified barriers according to a theoretical framework (the TDF), identifying 11 different barriers spanning four TDF domains: environmental context and resources (four barriers); knowledge and skills (four barriers); memory, attention, and decision processes (two barriers); and beliefs about capabilities (one barrier).

**Table 1:** Genetic referral barriers.

Barrier (coded to TDF domain)	Barrier descriptions
Knowledge	Provider lack of knowledge about genetics and/or referral criteria [3, 33, 35–38, 42–44, 48, 55, 58, 60, 61, 73]
	Provider lack of knowledge about the genetic counseling referral process [3, 34]
	Patient lack of knowledge about the availability of genetic services [35]
	Provider lack of knowledge about the availability of genetic services [34, 37, 73]
	Patient lack of knowledge about genetic testing and potential benefits [37]
	Provider lack of knowledge about criteria for pathology tests to guide genetic risk assessment [3]
	Lack of provider knowledge about the genetic counseling referral process [3, 34]
Skills	Difficulties communicating genetic information to patient [35]
	Limited exploration and/or documentation of patient family history [36, 44, 48, 54, 58]
	Incomplete ordering of pathology tests (e.g., mismatch repair immunohistochemistry) [3]
	Difficulties applying referral guidelines/criteria to identify patients at increased genetic risk [3, 55, 58]
	Training provided on an ad hoc basis, resulting in unfamiliarity with referral processes [3]
Environmental context & resources	Lack of on-site genetic counselors [34, 56]
	Limited availability of genetic counselors (due to clinical demands, limited resources) and subsequent long waitlists [34, 49, 56, 57]
	Lack of geographical access to genetic services [42, 49, 50]
	Patient financial constraints preventing access to genetic counseling and testing [50]
	Time required to collect a complete family history to assess genetic risk [51]
	Administrative referral barriers (e.g., referral forms not always available in clinic, faxing process can be fraught; multiple electronic management systems and departments with limited connectivity) [3]
	Delayed implementation of genetic screening tests (e.g., mismatch repair immunohistochemistry) [3]
	Lack of availability among genetic staff to attend multidisciplinary team meetings [3]
	Genetic risk assessment guidelines may be complex to interpret and unsuited to occasional use in a busy setting [38]
	Limited access to genetic services (reason not specified) [55]
	Time constraints and absence of primarily responsible pathologist act as a barrier to appropriate ordering of genetic screening tests [62]
	Limited integration of genetic testing into the cancer treatment workflow [56]
Memory, attention, & decision processes	Inconsistent documentation of referral recommendations [34]
	Difficulty deciding which patients were eligible for genetic screening tests (e.g., mismatch repair immunohistochemistry) [33]
	Difficulty deciding which patients warrant genetic referral [36, 43, 48, 55]
	Genetic risk assessment guidelines may be complex to interpret and unsuited to occasional use in a busy setting [38]
	Clinicians can easily forget to refer cases for genetic screening tests (e.g., microsatellite instability testing) [62]
	Interpreting pathology results can be difficult, making the decision-making process more difficult and less routine [3]
	Genetic referrals can be overlooked due to competing clinical priorities [3]
	Clinicians may not have the necessary information (e.g., immunohistochemistry reports) to make a decision about genetic referral [3]
Beliefs about consequences	Perception of limited clinical utility of genetic testing among providers [50]
	Lack of patient awareness about the potential benefits of genetic testing [37]
Beliefs about capabilities	Lack of confidence in ability to assess patients’ genetic risk or in providing genetic services [38, 42, 46]
	Terminology in the pathology reports can be confusing, generating the perception that it is hard to make an appropriate referral [3]
Emotion	Patient fear about the potential outcomes of genetic testing [37]
Social/professional role & identity	Lack of clarity about clinician roles in the genetic risk assessment process [33, 34]

*TDF* Theoretical Domains Framework.

### Intervention characteristics

The majority of articles described multicomponent interventions (*n* = 26; 81%), with 108 active intervention components identified across all included articles. More than half the intervention studies included provider education components (*n* = 17, 53%), most of which were delivered via face-to-face training (*n* = 10; 59%). Fifteen studies (47%) included family history and/or referral tools; of which ten (67%) also provided clinical decision-making support. Fifteen studies (47%) used clinical data review systems (e.g., review of pathology results for genetic referral indicators, discussion of clinical risk indicators at multidisciplinary team meetings). Remaining intervention components included (but were not limited to) clinician reminder systems (*n* = 10; 31%), embedding genetics staff within other clinical teams (*n* = 10; 31%), automated electronic referral systems (*n* = 6; 19%), development of clinical protocols and flowcharts (*n* = 5; 16%), and audit and feedback (*n* = 3; 9%). Intervention components are described in full in Supplementary File 4. While this review focused on interventions targeted at the health system or provider level, nine articles (28%) also incorporated patient-level intervention components, the majority of which were educational materials (*n* = 8; 89%) or involved patient completion of family history tools (*n* = 3; 33%).

### Implementation strategies

Less than half the articles (*n* = 15; 47%) explicitly described implementation strategies used to promote uptake of active intervention components. Thirty strategies (coded to the ERIC taxonomy) were identified, the majority of which (*n* = 25; 83%) took place at the preimplementation stage. Three studies [3, 33, 34] conducted formal assessment of local barriers and facilitators to tailor the design of intervention strategies, using methods such as stakeholder working groups [3, 34], environmental scans [33], and validated barrier questionnaires [3]. Eight studies [3, 35–41] conducted preimplementation meetings among stakeholder workgroups to plan the improvement effort and allow intervention refinement. Other preimplementation strategies included identifying and preparing local champions [39, 41, 42], conducting educational meetings about the intervention [40, 43–45], involving executive boards [3, 41], developing a formal implementation blueprint [40], and forming partnerships with implementation experts [3]. Interventions were designed by end users in five studies [33, 34, 37–39], while five studies used a co-design approach with collaboration between researchers and end users [3, 36, 40, 46, 47]. The remaining 22 studies did not specify the approach to intervention design.

Mid-implementation strategies included the provision of ongoing researcher support to promote intervention uptake [40, 44], tailoring of strategies based on clinician experiences [36, 43], and provision of auditing feedback on intervention adherence [45]. No postimplementation strategies were identified. Full descriptions of the implementation strategies are provided in Supplementary File 5.

### Application of theory and frameworks

Few studies (*n* = 4; 13%) explicitly made use of implementation science or behavior change theory frameworks in the design and/or implementation of intervention strategies. Applying the coding scheme developed by Michie et al. [29], only one article [3] applied behavior change theory to the full extent (e.g., describing how theoretical constructs were used to inform the design of each intervention strategy). In this article, the Theoretical Domains Framework Implementation (TDFI) provided a structured process through which local barriers (mapped to TDF domains) were identified, with the design of a suite of targeted intervention strategies informed by corresponding BCTs [3]. Of the remaining three studies [33, 34, 37], theoretical frameworks were only partially applied (e.g., broad description of how theory was used in intervention design).

Analysis of the 108 active intervention components across all studies identified 16 categories of BCTs, which were used on 214 occasions (intervention components often contained multiple BCTs). The most frequently occurring BCTs were *social support—practical* (examples included assisted interpretation of molecular screening tests, genetic counselor attendance at multidisciplinary meetings), which was used on 43 occasions across 22 studies; *prompts and cues* (e.g., referral reminder systems, electronic alerts) used on 38 occasions across 23 studies; *conserving mental resources* (e.g., clinical decision support tools) used on 29 occasions across 20 studies; and *information about health consequences* (e.g., educational workshops and resources about Lynch syndrome) used on 28 occasions across 14 studies. Only one study explicitly stated the BCTs used in intervention strategies [3], with the majority of studies requiring retrospective coding of BCTs based on intervention descriptions. Supplementary file 4 provides the full BCT mapping exercise.

After applying the Theory and Techniques Tool [30], nine BCTs were used to address a corresponding barrier that has previously demonstrated statistically significant mechanistic links (i.e., theoretical alignment agreed upon by expert consensus AND associations in the intervention literature synthesis). These were used on 83 occasions, identified within 59 intervention components (each intervention component can contain multiple BCTs) across 23 studies. The most frequently cited BCTs with evidence of mechanistic links were *information about health consequences* (TDF domain = knowledge), used on 20 occasions; *social support (practical)* (TDF domain = environmental context and resources). used on 19 occasions; *prompts and cues* (TDF domains = environmental context and resources and memory, attention, and decision processes), used on 17 occasions; and *instruction on how to perform the behavior* (TDF domains = knowledge and beliefs about capabilities), used on 12 occasions. Theoretical alignment was unable to be assessed for 37 of the 108 strategies (34%), due to insufficient descriptions of barriers and/or corresponding intervention strategies.

The BCT *social support—practical* was the most frequently observed BCT among studies that successfully led to improvements in referral (used on 32 occasions across 16 studies), while the BCT *information about health consequences* was the most frequently observed among studies that did not lead to improvements (used on 14 occasions across 5 studies). Among successful studies, 41% (41/99) of the examinable BCTs had evidence of mechanistic links according to the Theory and Techniques Tool [30], compared to 25% (11/44) in the nonsuccessful studies. Table 2 provides a compilation of example strategies with proven mechanistic links from successful studies, stating their proposed causal pathway.

**Table 2:** Example intervention strategies and their mechanistic links.

TDF domain (MoA)	Barrier description	Behavior change technique	Example intervention strategy	Reference
**Environmental context & resources**	Competing clinical demands make it difficult to ensure referrals are enacted for patients at increased hereditary cancer risk	**3.2 Social support (practical)**	Patient navigator role developed (cancer nurses) to assist with coordination of genetic referrals. Navigators kept lists of the tumor screen-positive patients as reported by pathologists, and helped to coordinate their referrals for genetic counseling.	Miesfeldt, 2018 [49]
	Limited integration of genetic testing into the cancer treatment workflow; long wait times for genetic testing, workforce shortages of genetic counselors (GC), and a lack of easily available genetic counseling in clinic locations	**3.2 Social support (practical)**	A dedicated genetic counselor was co-located in the medical and gynecologic oncology clinic provider workroom. The genetic counselor met with providers daily and was available to see patients in real time, as needed. The genetic counselor’s contact details were supplied to all providers and they were encouraged to reach out if there was any uncertainty about genetic testing.	Rana, 2020 [56]
	Lack of geographical access to genetic counseling, with no on-site genetics team	**12.5 Adding objects to the environment**	Patients at a remote site could complete a family history survey via computer tablet during their oncology treatment. Information is remotely accessible by an offsite genetics team to identify and triage eligible patients.	Cohen, 2013 [42]
**Knowledge**	Provider lack of understanding about the potential clinical utility of genetic testing (in the context of hereditary cancer)	**5.1 Information about health consequences**	Lecture series delivered to health-care providers on the topics of hereditary cancer risk assessment, genetic testing, hereditary breast–ovarian cancer, and hereditary colon cancer.	Scheuner, 2013 [36]
	Provider lack of knowledge about genetics and/or referral criteria	**4.1 Instruction on how to perform the behavior**	A “traffic light” classification system was developed for the most common cancer types seen in the unit, providing instruction on the indicators for genetic referral.	Moss, 2019 [58]
**Memory, attention, & decision processes**	Performing the recommended pathology genetic screening tests [e.g., microsatellite instability for colorectal cancer (CRC) cases can easily be forgotten	**7.1 Prompts & cues**	Pathologists were provided with monthly electronic reminders on patients meeting inclusion criteria for genetic screening.	Overbeek, 2020 [62]
	Numerous (sometimes conflicting) guidelines make it difficult for clinicians to decide which patients are eligible for genetic referral	**11.3 Conserving mental resources**	Patients entered relevant personal health and family history information into an electronic form, through which a report is generated summarizing the patients genetic risk and guiding clinicians about whether or not a genetic referral is indicated	Edelman, 2014 [40]
**Beliefs about capabilities**	Discomfort by nongenetics health-care providers in providing genetic services (lack of knowledge & confidence)	**6.1 Demonstration of the behavior**	A registered nurse shadowed a genetic counselor and attended genetic counseling sessions for observational purposes prior to offering nurse-led genetic risk assessment and testing	Cohen, 2013 [42]

### Study outcomes

#### Service level outcomes

Eighteen studies (56%) resulted in significant improvements in genetic referral practices. Among these studies, clinical data review systems [33, 41, 48–53], referral and family history tools [36, 37, 40, 43, 48, 51, 54], and efforts to embed genetic staff into nongenetics services [33, 52–57] were the most frequently cited intervention components. An additional five studies (17%) also showed improvements, though were not powered for significance [34, 39, 42, 47, 58]. Two studies had mixed results (e.g., improvements in some referral outcomes but not others) [3, 40, 59], while changes in referral rates could not be established in one study due to lack of historical comparator [60]. Five studies (16%) showed no significant improvement in referral [35, 38, 44, 46, 61]. Individual quality appraisal scores are provided in Supplementary File 3. Using the GRADE assessment tool [32], there were some concerns about the certainty of the evidence across studies given the potential for publication bias, and since most studies used observational cohort design with historical controls (many of which did not report controlling for potential confounders, e.g., changes in genetic testing and referral guidelines over time).

#### Implementation outcomes

Twelve studies (38%) incorporated implementation outcomes in their reporting, though none formally applied the Proctor framework. These included adoption (*n* = 8; 25%), acceptability (*n* = 6; 19%), appropriateness (*n* = 4; 13%), feasibility (*n* = 3; 9%), and cost (*n* = 2; 6%). The extent to which health-care professionals adopted the planned intervention strategies ranged from 15% [38] to 67% [35]. For example, in a study implementing a software program to facilitate assessment of familial cancer risk in general practice, only 14% of the practitioners surveyed had used the program at least once in the one-year study period (potentially explaining the lack of improvement in genetic referral rates) [38]. Clinicians varied in their beliefs about the appropriateness, acceptability and feasibility of interventions, for example clinicians in three studies [3, 48, 60] found the interventions too time-consuming for day-to-day use. Of the two studies reporting economic outcomes [38, 62], these were expressed as marginal costs (e.g., unit costs associated with active intervention components, without reporting of costs to implement). Three studies [3, 40, 43] were accompanied by process evaluations (reported separately) providing in-depth insights on implementation outcomes and contextual factors potentially impacting implementation success.

## DISCUSSION

The speed at which genetic research is evolving requires ongoing evidence-based interventions to ensure meaningful translation into clinical practice. However, this systematic review has highlighted the variable success of intervention efforts to improve genetic referral, and the limited extent to which implementation science theories and frameworks have been applied to date. To advance the science of implementation in genetics and genomics, this review has comprehensively consolidated existing interventions according to standardized terminology, demonstrating patterns of successful and less successful approaches.

Despite calls for the explicit use of theory to better understand behavior change determinants, identify mechanisms of impact, and inform intervention strategies [17], this review has highlighted the underutilization of theory to address genetic referral practice gaps. This is comparable to findings from two systematic reviews of genomics intervention studies, in which both authors noted an absence of studies incorporating behavior change frameworks [63, 64]. Outside the genetics setting, studies have also demonstrated limited use of theory to address implementation challenges or guide intervention design [19]. Health-care professionals may not be aware that such frameworks exist, or require additional training and support to apply them in a meaningful and practical way [17, 20]. Efforts are currently underway to upskill clinicians and researchers to be able to apply these frameworks in the design of evidence-based intervention strategies [65, 66].

While recognizing the potential value of theory and implementation science frameworks in the genetics setting, a number of intuitively designed interventions (i.e., those designed by clinicians without explicit use of theory) in this review were successful in achieving significant improvements in genetic referral practices. While the use of this “informal theory” can be effective in producing positive practice change, explicit application of theory serves to promote learning across a range of studies, contexts, and clinical problems so as to avoid reinventing the wheel [17]. Furthermore, while intuitive and common-sense approaches can often be used to solve basic problems in ways that retrospectively align with theory (e.g., training opportunities to address knowledge and skill barriers), formal and prospective application of theory may also serve to better understand and address more complex barriers (e.g., emotion, social influences) that require more sophisticated approaches for tailored intervention design. A co-design model—harnessing the strengths of health-care professionals and behavior change experts—may support the development of intervention strategies that are both theoretically informed *and* fit for context [67].

Although the ideal is to apply theory prospectively in the design of intervention strategies, retrospectively coding intuitive interventions may help to understand the processes by which these strategies produce their effects, while also optimizing the design of future intervention efforts to address genetic referral practices. Through this review, we have consolidated a large number of intervention strategies matched to key psychosocial barriers, and demonstrated the extent to which these barriers and corresponding intervention strategies align with TDF domains and corresponding BCTs. We have also compiled a list of example strategies with proven mechanistic links (across a range of barrier domains) that have been successfully used in studies that resulted in improvements in genetic referral practices. In addition to demonstrating how BCTs can be operationalized and theoretical links made explicit, these provide a resource for clinicians embarking on future efforts to improve genetic referral practices, allowing potential selection of strategies with existing mechanistic links to address similar psychosocial barriers. This is a relatively novel strategy, which, to our knowledge, has been previously applied only once in the context of intuitive genetic counseling interventions aimed at improving family communication of genetic cancer risk information [20]. The process was found by clinicians to be highly valuable in developing a practical understanding of the application of behavior change theory [20].

In this systematic review, knowledge was the most frequently cited barrier to genetic referral practices. This is consistent with other systematic reviews that have used the TDF to categorize behavioral determinants surrounding various aspects of genetic practice—one in the context of nongenetic specialist decision-making about offering genetic and genomic testing [63], and another related to mainstreaming of genetics and genomics for nurses and physicians [68]. While it is useful to gain a collective understanding of the commonly experienced barriers to genetic referral practices, barriers are highly context-specific and can vary widely across different settings. It is therefore crucial to gain an in-depth understanding of local barriers to ensure the design of intervention strategies targeted to the local context. Despite the availability of multiple systematic approaches to identify context-specific determinants and match appropriate intervention strategies [19], only three studies in this review conducted local barrier assessments at the preimplementation stage [3, 33, 34]. Assumption of barriers, even when guided by literature reports, may lead to selection of strategies that are not contextually relevant, and hence less likely to be effective. For example, two studies in this review focusing on educational strategies (based on the assumption of clinician knowledge barriers) demonstrated no improvements in referral practices despite increases in knowledge [35, 61], suggesting involvement of other determinants not targeted for change. Such findings may also suggest that increasing knowledge alone is insufficient for achieving behavior change. To address these issues, theory-guided approaches can elicit a more meaningful and accurate representation of barriers to better inform intervention design [15].

Relatively few studies in this review were accompanied by formal process evaluations, or reported on implementation outcomes. While previous systematic reviews of health system interventions have rarely included formal measurement and reporting of these outcomes, similar findings have been demonstrated in the cardiovascular intervention setting [69]. Consequently, when studies fail to achieve the desired clinical outcome (in this case, improvements in genetic referral practices) researchers and clinicians are unable to distinguish between interventions that are inherently faulty in their design, versus those poorly implemented [70]. To address this issue, the UK MRC has provided a comprehensive statement to guide researchers in the design of process evaluations to maximize the interpretation of complex intervention trials [22].

Among studies that reported on implementation outcomes, a number cited suboptimal adoption as a potential explanation for lack of improvement in genetic referral [35, 38, 44]. In such instances, real-time process evaluation data can shed light on the factors underlying suboptimal implementation, allowing interventions to be tailored and adapted to the needs and preferences of end users [71]. In this review, only two studies provided opportunities during the implementation phase to adapt strategies to ensure a better fit for the local context [36, 43]. While intervention adaptations were once broadly perceived as a threat to fidelity (and therefore study quality and effectiveness), there is growing recognition that adaptations may in fact have a positive impact on study outcomes—provided that the intended underlying causal mechanisms (i.e., core functions) are maintained [71]. Such approaches can also facilitate scale-up efforts, though require an in-depth understanding of the underlying theory and causal processes through which the intervention is intended to produce effects.

Finally, very few studies in this review incorporated measures to assess the economic impact of intervention implementation [38, 62]. For policymakers and health managers to justify the allocation of resources for implementation efforts, there is a need not only to establish the clinical effectiveness (i.e., improvements in referral rates), but also cost-effectiveness [72]. To ensure efficient use of health system resources, formal economic evaluations are needed (incorporating costs of both intervention and implementation strategies) to determine the most cost-effective implementation approaches [72].

## Limitations

To assess intervention effectiveness, papers were included only if they reported at least one clinical or service level outcome representing genetic referral practices (e.g., genetic referral rates, genetic counseling attendance, uptake of genetic tests). Papers reporting only on nonclinical outcomes (e.g., clinician knowledge, confidence, self-reported referral practices) were excluded. Therefore, insights gained from this review may not be representative of the broader body of intervention research in this area. Furthermore, the majority of interventions took place in the cancer setting, and therefore may not be generalizable to other genetic subspecialties.

Although full texts were independently assessed by two reviewers, extraction was predominantly performed by a single reviewer. Although a second reviewer independently extracted data for 20% of randomly selected studies, there remains some potential for error in the extraction process. As noted in the results, there were also potential biases both within and across studies, and findings from this systematic review should therefore be interpreted with caution. Coding of barriers, implementation approaches, and intervention strategies were limited by the extent to which these were adequately described within studies, with many providing insufficient detail to enable coding. Results of the coding exercise should also be interpreted with caution. This highlights the need for better reporting of interventions, particularly given that no included studies explicitly made use of available intervention reporting guidelines (acknowledging that some studies were published before the guidelines were introduced) [24, 25]. Standardized reporting would also enable opportunities for meta-analyses, with the potential to quantify the significance of theoretical contributions to referral interventions for producing better outcomes.

### Conclusions

Given the speed at which genetics and genomics is evolving, the application of implementation science and/or behavior change methods is crucial for ensuring smoother translation, while advancing current understanding about what strategies work, and why. However, this review has demonstrated that the use of such methods in the context of interventions aimed at improving genetic referral practices is lacking. Further efforts to upskill genetic health-care professionals to apply these methods in practice, and/or to build collaborative partnerships with implementation science and behavior change experts are needed. In light of findings from this review, we propose the following recommendations for consideration when designing, implementing, and reporting future intervention efforts in the genetics setting:

- A thorough, theory-guided assessment of local barriers conducted at the preimplementation stage to inform the design of targeted, context-specific intervention strategies
- Utilization of theories, models, and frameworks to optimize the design of genetic intervention strategies and opportunities for successful implementation, and support a standardized understanding of effective ingredients for eliciting change
- Formal assessment of implementation outcomes to enhance interpretation of clinical outcomes, providing an understanding of the factors impacting success
- Carefully designed process evaluations conducted alongside intervention studies can provide an in-depth understanding of what works (or does not) and why, while economic evaluations can guide decisions about resource allocation for future implementation and/or scale-up efforts
- Process evaluations can also provide opportunities to adapt interventions to ensure better fit for context, provided that the proposed causal pathways are maintained
- When reporting intervention studies, reporting guidelines (such as StaRI and TIDieR) should be utilized to enhance intervention replicability, with underlying theory made explicit

In addition to maximizing opportunities for successful implementation, applying the above recommendations will serve to promote learning across interventions to address clinical problems within and beyond genetics to ensure smoother translation of new (and existing) evidence into practice.

## Supplementary information

Supplementary file 1

SUPPLEMENTARY FILE 3

SUPPLEMENTARY FILE 4

SUPPLEMENTARY FILE 5

## Data Availability

Data and materials (e.g., template data collection forms) are available upon written request.
