# Supplementary material for: The design, implementation, and effectiveness of intervention strategies aimed at improving genetic referral practices: a systematic review of the literature
Source: Genet Med. 2021 Aug 24;23(12):2239–49. doi: 10.1038/s41436-021-01272-0 (PMC8629749; doi:10.1038/s41436-021-01272-0)
Supplement: Supplementary file 1 [file 41436_2021_1272_MOESM1_ESM.pdf]

Supplementary file 1. Search terms

#	Searches
1	(genetic clinic* adj3 (referral* or referred or referring)).tw.
2	(genetic counsel* adj3 (referral* or referred or referring)).tw.
3	(genetic service* adj3 (referral* or referred or referring)).tw.
4	(genetic test* adj3 (referral* or referred or referring)).tw.
5	((famil* or heredit* or inherit*) adj3 (counsel* or service* or clinic*)).tw.
6	exp genetic counseling/
7	exp genetic service/
8	genetic testing/ or genetic carrier screening/
9	(referral* or referred or referring).tw.
10	5 or 6 or 7 or 8
11	9 and 10
12	1 or 2 or 3 or 4 or 11
13	limit 12 to english language
14	limit 13 to human
15	limit 14 to yr="1998 -Current"
16	limit 15 to yr="1998 - 2010"
17	limit 15 to yr="2011 - 2015"
18	limit 15 to yr="2016 -Current"
19	remove duplicates from 16
20	remove duplicates from 17
21	remove duplicates from 18
22	19 or 20 or 21

Supplementary File 2 - PICO

What are the impacts of interventions aimed at improving patient referral to genetic services?

Population	Intervention	Comparator	Outcomes
Health system<br>Health service<br>Health professionals	Any implementation intervention aimed at improving patient referral to specialist genetic services	Pre-intervention referral rates within same group; control group not exposed to intervention (e.g. standard practice); other active intervention	*Clinical outcomes:*<br>• Changes in genetic referral rates<br>• Surrogate outcomes for referral (e.g. genetic attendance rates or genetic testing uptake)<br>*Implementation evaluation outcomes*<br>• Proctor outcomes<br>*Cost-effectiveness:*<br>• *ICER/QALY/DALY/costs*

**Not required for intervention outcomes*; DALY = disability-adjusted life year; ICER = incremental cost-effectiveness ratio; QALY = quality-adjusted life year*

Research sub-questions:

- To what extent are implementation theories and frameworks applied in the design of interventions aimed at improving referral to genetic services?
- To what extent is process evaluation data collected to explain intervention outcomes

SELECTION CRITERIA

Selection criteria	Inclusion criteria	Exclusion criteria
Study type	Implementation intervention studies	
Study design	Studies with comparators: RCTs, cohort studies (concurrent or historical controls)<br><br>Qualitative studies or case series for implementation evaluation outcomes (Proctor outcomes)	Studies lacking outcome data (e.g. protocols)<br>Studies lacking comparator
Setting	Australia, NZ, Europe, Canada, USA (given similarities in genetic referral and testing guidelines)	Studies outside Australia, NZ, Europe, Canada, USA
Population	Health system or service<br>Health professionals<br>Patients (where at least one intervention component targets a health professional group or health service/system).	Patient only interventions
Intervention	Implementation intervention or intervention with implementation intervention component designed to improve referral to genetic services	Interventions that do not contain an implementation intervention component (e.g. technical intervention only)<br>Mainstreaming interventions<br>Interventions not aimed at improving genetic referral<br>Interventions implemented before Jan 1st 2000
Comparator	Standard/usual care<br>Another implementation intervention<br>Pre-intervention (baseline)	No comparator - for health system, implementation or cost-effectiveness outcomes
Outcomes	*Clinical outcomes:*<br>• Changes in genetic referral rates<br>• Surrogate outcomes for referral (e.g. genetic attendance rates or genetic testing uptake)<br>*Implementation evaluation outcomes*<br>• Proctor outcomes<br>*Cost-effectiveness:*<br>• *ICER/QALY/DALY/Costs*	No relevant health system or implementation evaluation outcomes
Language	English	Not in English
Publication period	From January 1st 2000	Before January 1st 2000
Publication type	Journal article	Conference proceedings, protocols, comments, letters, news, editorials, narrative, rapid or scoping reviews, theses
